# iPcc: a novel feature extraction method for accurate disease class discovery and prediction

**DOI:** 10.1093/nar/gkt343

**Published:** 2013-06-12

**Authors:** Xianwen Ren, Yong Wang, Xiang-Sun Zhang, Qi Jin

**Affiliations:** ^1^MOH Key Laboratory of Systems Biology of Pathogens, Institute of Pathogen Biology, Chinese Academy of Medical Sciences and Peking Union Medical College, Beijing 100730, China, ^2^Academy of Mathematics and Systems Science, Chinese Academy of Sciences, Beijing, 100190, China and ^3^National Center for Mathematics and Interdisciplinary Sciences, Chinese Academy of Sciences, Beijing, 100190, China

## Abstract

Gene expression profiling has gradually become a routine procedure for disease diagnosis and classification. In the past decade, many computational methods have been proposed, resulting in great improvements on various levels, including feature selection and algorithms for classification and clustering. In this study, we present iPcc, a novel method from the feature extraction perspective to further propel gene expression profiling technologies from bench to bedside. We define ‘correlation feature space’ for samples based on the gene expression profiles by iterative employment of Pearson’s correlation coefficient. Numerical experiments on both simulated and real gene expression data sets demonstrate that iPcc can greatly highlight the latent patterns underlying noisy gene expression data and thus greatly improve the robustness and accuracy of the algorithms currently available for disease diagnosis and classification based on gene expression profiles.

## INTRODUCTION

With the rapid development of high-throughput technologies, gene expression profiling based on microarrays or next-generation sequencing techniques have been widely applied in clinical research (1–9). The big advantage of simultaneously measuring the expression levels of thousands of genes facilitates informative and accurate disease diagnosis and classification. However, it also incorporates many irrelevant genes, producing a feature vector with extremely high dimensionality (10,11). The situation is much exacerbated by sample limitations for algorithmic training, leading to the curse of dimensionality problems.

To surmount the difficulties caused by the curse of dimensionality problem, various approaches have been proposed and developed on different levels of the experimental and analytical protocol. On the experimental level, increasing the number of samples involved in the studies to enhance the statistical power and measuring the expression levels of a set of selected genes involved in a certain signaling pathway are two possible solutions. However, increasing the number of samples is generally difficult because of eligible sample limitations and the huge cost of high-throughput measurements. Furthermore, measuring genes involved in only selected pathways can introduce artificial biases. In fact, the rapid development of high-throughput technologies produces more and more information about samples, allowing unbiased investigation of the molecular truth of various biomedical phenomena. For example, RNA-Seq (deep sequencing the transcriptomes of samples) can detect expression levels of novel genes that are not annotated in the reference genomes, compared with the traditional gene expression profiling microarrays (8).

On the analytical level, adapting and developing algorithms capable of handling high-dimension data sets, computationally selecting a small set of relevant genes from the huge gene list for subsequent diagnosis and prognosis and extracting a small set of virtual genes that are certain functions of the real genes, are representative computational schemes to bypass the curse of dimensionality. In view of machine learning, these three schemes correspond to developing new algorithms of clustering and classification, feature selection and feature extraction. Until now, many algorithms suitable for dealing with high-dimension data sets [e.g. support vector machine (12–14), random forest (15–17), naïve Bayes classifier (18–20) and *k*-means (21–24)] have been applied to disease sample classification and clustering based on gene expression profiles. Various feature selection methods implemented by mathematical programming (25), Bayesian inference (26,27), ant colony algorithm (28) and mutual information (29,30) have been proposed and developed, facilitating accurate identification of relevant disease genes. The concept of virtual genes or meta-genes based on principle component analysis and nonnegative matrix factorization was introduced from the feature extraction perspective to reduce the number of dimensions of the data sets in the meta-gene space (31–34). These methods, based on new clustering/classification algorithms, gene selection and virtual genes, greatly improve the accuracy of disease diagnosis and prognosis on gene expression measurement but neglect an important feature space formed by sample correlations.

In this study, we propose a novel method to negotiate the dimensionality problem in clinical gene expression studies. Similar to the available feature extraction methods, we define a series of novel features for samples based on the gene expression profiles by iterative use of Pearson’s correlation coefficient. These features form a feature space, referred to as ‘correlation feature space’. We demonstrate that the latent structures between samples can be easily highlighted based on the newly defined features, even though many irrelevant genes are included in the original data. Furthermore, we illustrate that the newly defined features can greatly improve the accuracy of the currently available clustering and classification algorithms.

In current biomedical studies, two typical strategies are widely used to analyze gene expression data for clinical purposes: class discovery and class prediction (5). Class discovery tries to discover new disease subtypes based on gene expression patterns, while class prediction tends to assign particular samples to well-defined disease classes. Both strategies have significant potential to improve cancer diagnosis, prognosis and therapies. On real prostate cancer, leukemia and psoriasis data sets, we demonstrate that the newly defined features greatly leverage the power of the current class discovery and class prediction methods. Therefore, we propose a novel type of noise-resistant features based on iterative Pearson correlation coefficients, which is substantially helpful to boost gene expression profiles from bench to bedside.

## MATERIALS AND METHODS

### Sample correlation feature space defined by gene expression profiles

Given a gene expression data set 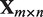, in which the expression of *n* genes is measured for *m* samples and 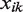 denotes the expression level of gene *k* in sample *i*, we first calculate 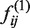, the correlation of the gene expression profiles of samples *i* and *j*, by the Pearson correlation coefficient (35):
(1)
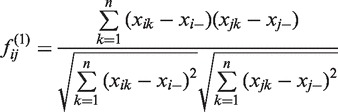

where 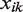 and 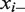 are the expression level of gene *k* and the average gene expression level of sample *i*, respectively. The variables 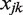 and 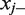 are the expression level of gene *k* and the average gene expression level of sample *j*, respectively. Finally, we obtain a symmetric matrix 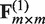 in which 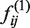 is its element. Function 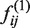 measures how the gene expression profiles of samples *i* and *j* correlate to each other. However, here we interpret it as a feature of sample *i* that is scored by sample *j*. Based on this interpretation, 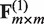 becomes a matrix of *m* samples and *m* features. Thereafter, we can define 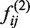 as follows:
(2)
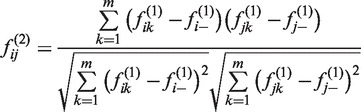



We name 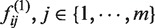 as the first-order correlation features of sample *i* and 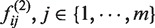 as the second-order correlation features of sample *i*. Generally, we can define the *t*-order correlation features of sample *i* as follows:
(3)
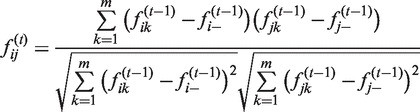



All the features 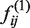, 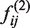, … , 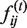, … , form a correlation feature space.

Because the correlation features are defined based on Pearson correlation coefficients, we named the computational method iterative Pearson correlation coefficients (iPcc). It can be seen that correlation features have several obvious properties: (i) 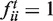 for any *i* and *t*; (ii) 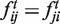 for any *i*, *j* and *t*; and (iii) 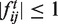 for any *i*, *j* and *t*. Below we compose three toy gene expression data sets of six genes (for example) to demonstrate numerically other properties of sample correlation features in an oversimplified setting.

**Figure 1.**
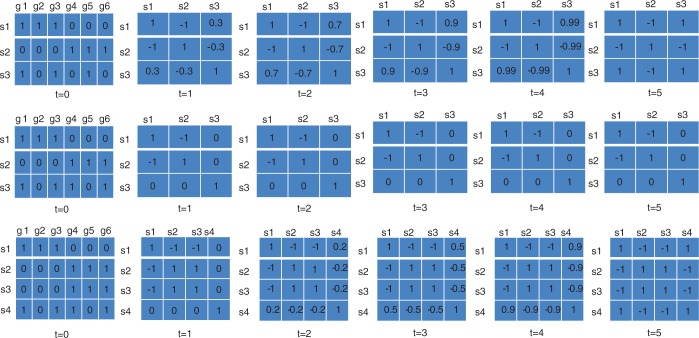
Three toy examples demonstrate correlation features generated by iPcc. In the top line, three samples are depicted by six virtual genes in which s1 and s2 have distinct gene expression profiles, and s3 has a gene expression profile similar to that of s1. Based on the original features (*t* = 0), the first- (*t* = 1), second- (*t* = 2), third- (*t* = 3), fourth- (*t* = 4) and fifth-order (*t* = 5) correlation features were constructed by iPcc. The fifth-order correlation features suggest that s3 belongs to the s1 class. In the middle line, s1 and s2 are the same as those in the top line, whereas s3 is similar to neither s1 nor s2. Those constructed correlation features suggest that s3 is similar to neither s1 nor s2 even with higher orders. In the bottom line, s1 and s2 are still the same as in the top and middle lines, s3 is the same as s2, and s4 is the same as s3 in the middle line. Based on the first-order correlation features, s4 is found to be similar to neither s1 nor s2 and s3. However, on the second-order correlation features, s4 is found to be weakly similar to s1. Increasing the order to the fifth order, s4 is found to have the same fifth-order correlation features as s1, suggesting that iPcc can reveal sample similarity hiding in high-order correlation features.

In toy Data set 1, three samples are composed in which samples 1 and 2 (s1 and s2) represent two different classes, whereas sample 3 (s3) is more similar to sample 1 (Figure 1). By calculating the first-order correlation features of these three samples, we can observe that s3 has a correlation feature vector of (0.3, −0.3, 1) that is similar to that of s1, which is (1, −1, 0.3). By calculating the second-order correlation features, the feature vector of s3 becomes (0.7, −0.7, 1) while the feature vector of s1 is (1, −1, 0.7). When the fifth-order or higher correlation features are calculated, the feature vectors of s3 and s1 become the same, whereas the feature vectors of s3 and s2 become opposite. In other words, the similarity of s3 and s1 measured by Pearson correlation coefficients based on their features converges from 0.3 to 1, while the similarity of s3 and s2 converges from −0.3 to −1. Thus, in this oversimplified setting, 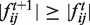 holds true.

In toy Data set 1, the initial feature profile of s3 is closer to that of s1, and the high-order correlation feature profile of s3 converges to the same as that of s1, suggesting that correlation features can enlarge weak pattern underlying the raw data set. In toy Data set 2, we demonstrate that correlation features can also preserve independent relationships among samples. We compose an initial feature profile for s3 that has equal distance to those of s1 and s2. The first-order correlation feature vector of s3 is (0, 0, 1), which is vertical to both of the feature vectors of s1 and s2. The second-order or higher correlation feature vectors of s3 are the same as the first-order correlation feature vector of s3, suggesting that the independence of s3 from s1 and s2 is conserved during the iterative correlation feature extraction.

In toy Data set 3, we conceive two different classes of unbalanced class sizes (s1 for Class 1, s2 and s3 for Class 2). We further conceive s4 that has equal distance from s1, s2 and s3. The first-order correlation feature vector of s4 is (0, 0, 0, 1), suggesting that s4 forms an independent class from s1, s2 and s3. However, the second-order correlation feature vector of s4 indicates weak similarity to that of s1. The higher-order correlation feature vectors of s4 highlight the similarity of s4 to s1 until their Pearson correlation coefficient converges to 1. Thus, correlation features not only can enlarge the weak patterns underlying the raw data set and preserve the balanced independent relationships, but also can highlight those patterns hidden in the higher feature space.

### Simulated and real gene expression data sets

We evaluated our method on both simulated and real gene expression data sets. First, we simulated a series of artificial gene expression data sets to show the properties and performance of the correlation features against the magnitude of uncertainty of gene expression measurements and the ratio of irrelevant genes to relevant genes. We designed three different sample classes. For each sample class, we generated two relevant genes with expression levels randomly sampled from a normal distribution with mean 1 and given a standard deviation *σ* from 0.1 to 0.5. We simulated 0-, 10-, 50- and 100-fold irrelevant genes according to a normal distribution with mean 0 and the same standard deviation as those six (2 × 3) relevant genes and added into the data sets. For each simulated data set, we applied our method and demonstrated the sample relationship by visualizing the sample similarity matrices in Figure 1. Clustering and classification algorithms were used to show the benefits produced by correlation features. We also evaluated iPcc on completely random data sets. We simulated three types of completely random data sets of 150 genes and 150 samples by sampling from the standard normal distribution, the uniform distribution on the interval [0, 1] and a discrete uniform distribution on {−1, 0, 1}. These completely random data sets provide negative examples to demonstrate the behavior of iPcc in situations no relationships exist.

To evaluate the advantage of correlation features to real-world applications, we examined the clustering and classification performance of a series of widely used algorithms on three real gene expression data sets. The first real gene expression data set was the gene expression data set of leukemia (5) in which 25 acute myeloid leukemia (AML) samples, 38 B-cell acute lymphoblastic leukemia (ALL) samples and 9 T-cell ALL samples were profiled genome-wide by gene expression microarrays. Among the 72 samples, 7129 probes were available. After removing probes with missing values, three preprocessing steps, including flooring/ceiling, filtering and log10-transformation as described in (36), were applied to select informative probes, resulting in 3571 informative probes. Thus, it is a typical gene expression data set with the curse of dimensionality.

The second data set was a prostate cancer gene expression data set (37) downloaded from the NCBI GEO database (38) with accession number GDS3289, which includes 104 samples and 9483 genes. Among the 104 samples, there are six types of samples: 22 normal samples from benign epithelial cells, 5 prostate cancer samples with atrophic lesions, 20 metastatic prostate cancer samples, 32 localized prostate cancers, 13 prostate cancer samples with prostatic intraepithelial neoplasia and 12 normal samples from benign stromal cells. Accurately revealing sample relationships by either clustering or classification algorithms from the large number of gene expression profiles is important for clinical diagnosis and even therapeutics.

The third data set contains two series of gene expression profiles of normal and psoriasis samples, downloaded from the NCBI database with accession numbers GSE13355 and GSE14905 (39,40). There are 180 samples in GSE13355 (122 normal samples and 58 psoriasis samples) and 82 samples in GSE14905 (49 normal samples and 33 psoriasis samples). In total 54 676 probes were applied to measure the expression levels of human genes. Because the two series of gene expression profiling experiments were conducted in different laboratories, batch effects exist. We used this data set to demonstrate how correlation features reveal the existence of batch effects and how batch effects are removed.

### Computational methods for disease class discovery and prediction used in this study

Besides the direct application of iPcc to the gene expression data sets to reveal the underlying patterns between samples, we also illustrate the leverage impact of iPcc on disease class discovery and prediction. For each data set, we used *k*-means to implement disease class discovery. *k*-means is a classic clustering algorithm that partition *n* samples into *k* clusters in which each sample belongs to the cluster with the nearest mean (24,41). *k*-means has been applied widely to gene expression data analysis, and various variants have been proposed. Because the computation of *k*-means is NP-hard, efficient heuristic algorithms are commonly used and generate local optima. Due to the curse of dimensionality caused by the high number of dimensions in gene expression data sets, *k*-means can give out different local optima during different runs in general. We demonstrate that iPcc can circumvent the curse of dimensionality and greatly enhance the power and robustness of *k*-means on gene expression data sets. This feature extraction method can also greatly improve the performance of supervised algorithms for disease class prediction. We take the naïve Bayes classifier (41) as an example to illustrate how iPcc boosts the accuracy of disease class prediction based on gene expression profiling. Finally, we show the great power of iPcc to remove batch effects in gene expression analysis.

## RESULTS

### iPcc highlights latent structures between samples with noisy simulated gene expression profiles

**Figure 2.**
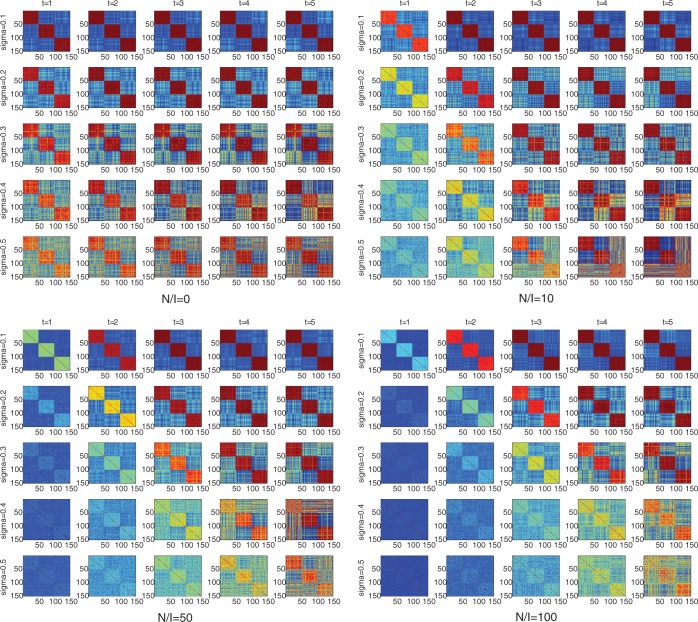
iPcc highlights patterns embedded in simulated samples with no, 10-, 50- and 100-fold noisy genes. Three sample classes were simulated, in which samples 1–50 belong to one class, samples 51–100 belong to another class and samples 101–150 belong to the third class. N/I denoted the ratio of the number of noisy genes to the number of informative genes. Sigma is the standard deviation of gene expression during simulation. ‘*t*’ is the order of correlation features.

We first tested the performance of iPcc on the simulated data sets. For the first simulated data set, which has no irrelevant genes, we observed that iPcc can reflect the true sample relationship faithfully with any order of correlation features, the same as the performance of the original features (Figure 2). After adding 10-fold noisy genes (the second simulated data set), we noted that the sample relationship revealed by the original features became more and more obscure when increasing the standard deviation *σ*. However, iPcc highlighted the latent sample relationship through interrogating the correlation features. When *σ* was 0.1, 0.2 and 0.3, iPcc perfectly unraveled the predefined sample relationship. As the order of correlation features increased, the patterns between samples became clearer and clearer (Figure 2). When *σ* was 0.4 or 0.5, there was no obvious pattern to be observed based on the original features. However, as the order of correlation features increased, the patterns underlying those samples were highlighted (Figure 2), although the highlighted patterns deviated a bit from the predefined sample similarity structure. This deviation was caused by the big *σ*, which redefined the sample similarity structure.

The potential of iPcc to highlight latent sample relationships beneath the high-dimension gene expression data was further demonstrated by increasing the number of noisy genes. In the third simulated data set, 50-fold noisy genes were added. The original features only revealed weak similarity between samples from the same predefined class at *σ* = 0.1 and 0.2. When *σ* = 0.3, 0.4 or 0.5, the predefined sample classes could not be reflected by the original features at all (Figure 2). However, as the order of correlation features increased, the sample relationships at *σ* = 0.1 and *σ* = 0.2 were enlarged, and the sample relationship at *σ* = 0.3 was unraveled almost perfectly. Although the predefined sample structure was not precisely reproduced at *σ* = 0.4 or 0.5, it was dug out from the vagueness.

When the number of noisy genes was 100-fold the number of informative genes (the fourth simulated data set), the original features could only reveal weak correlations between samples from the same classes at *σ* = 0.1, and the sample patterns were blurred at *σ* = 0.2, 0.3, 0.4 and 0.5. When *σ* = 0.3, 0.4 or 0.5, there were no obvious similar samples observed (Figure 2). However, iPcc reproduced the sample relationship almost completely at *σ* = 0.1, 0.2 and 0.3 and unraveled the underlying sample patterns at *σ* = 0.4 and 0.5 with a slight deviation from the predefined sample classes.

### iPcc improves the accuracy of clustering and classification algorithms on noisy simulated gene expression profiles

**Figure 3.**
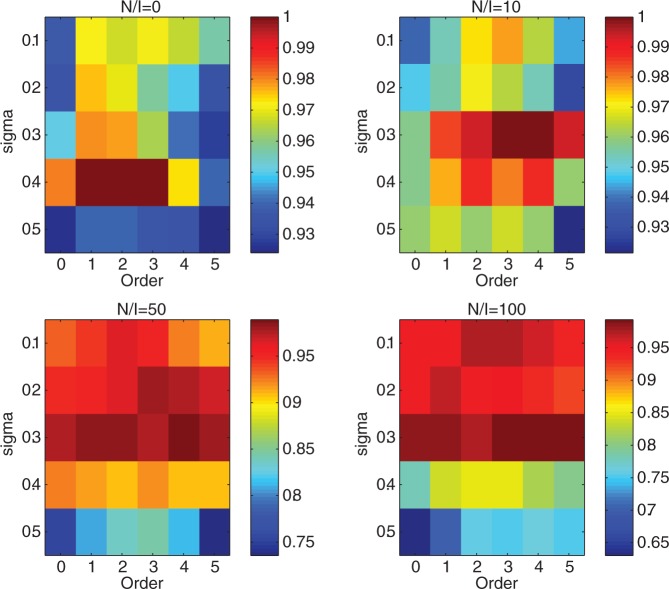
Average clustering accuracy of 1000 *k*-means (*k* = 3) runs on the simulated data sets with various noisy gene levels and orders of correlation features. Order 0 signifies the original simulated gene expression profiles.

We tested the clustering and classification algorithms *k*-means and the naïve Bayes classifier on the simulated data sets. For all four simulation data sets with 0-, 10-, 50- and 100-fold noisy genes, *k*-means generated more accurate clustering results (average accuracy in 1000 runs) in general based on the correlation features than those based on the original features (Figure 3). When *σ* = 0.1, the average accuracy of *k*-means on the original four simulated data sets was ∼93%, whereas the highest accuracy of *k*-means on the correlation features of the four data sets reached ∼97%. When *σ* increased, the trends still existed and the accuracy increment could even reach 12% (100-fold noisy genes).

We tested the performance of the naïve Bayes classifier by leave-one-out cross-validation evaluations on the four simulated data sets. The naïve Bayes classifier generated more accurate classification results on the correlation features (with order 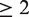) than those of the original features. When *σ* = 0.1, 0.2 or 0.3, the prediction accuracy approached 1 on both the original features and correlation features for all four simulated data sets. When *σ* = 0.4 or 0.5, the prediction accuracy increased from 86 to 90% when 10-fold noisy genes were added, from 88 to 90% when 50-fold noisy genes were added and from 71 to 80% when 100-fold noisy genes were added.

The performance of iPcc on the completely random data sets suggested that the clustering and classification accuracy based on those correlation features generated by iPcc was similar to that on the original features. For each type of statistical distributions, we generated 1000 completely random data sets. For each data set, we calculated the clustering (by *k*-means) and classification (by the naïve Bayes classifier) accuracy on both the original features and the correlation features. We applied the Student’s *t*-test to those accuracy values and found that no significant differences were observed between the original features and the correlation features for both clustering and classification.

### iPcc leverages the power of algorithms for disease class discovery and prediction on a real leukemia data set with two classes

**Figure 4.**
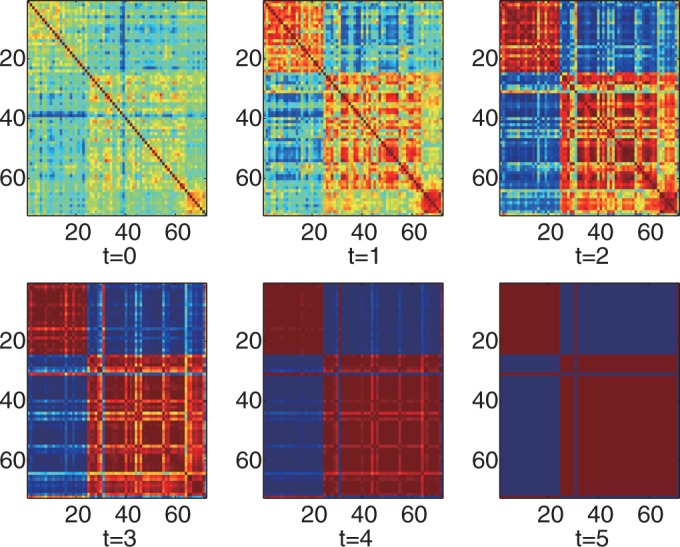
Heatmaps of the leukemia sample relationships on the original, first-, second-, third-, fourth- and fifth-order correlation features. Samples 1–25 are AMLs. Samples 26–63 are B-cell ALLs. And samples 64–72 are T-cell ALLs.

We further tested the performance of iPcc on real gene expression data sets for three different types of diseases: leukemia, prostate cancer and psoriasis. First, we evaluated the sample relationship reflected in the whole gene expression profiles of the leukemia data set (3571 probes in total after preprocessing) by iPcc. The sample similarity matrix revealed by the original 3571 features weakly suggest the existence of two big groups: the AML group (samples 1–24) and the ALL group (samples 25–72), in which sample 25 was wrongly indicated and is in fact an AML sample (Figure 4). The ALL group was further suggested weakly to be composed of two small subgroups: the B-cell ALL group (samples 25–63) and the T-cell ALL group (samples 64–72). The average intra-class sample similarity (AML–AML and ALL–ALL similarities) was 0.7262, while the average inter-class sample similarity (AML–ALL similarities) was 0.6572.

However, the sample similarity matrix constructed based on the first-order correlation features by iPcc strongly indicated the existence of the AML and ALL groups (Figure 4). In the sample similarity matrix constructed of the original features, the difference between the average intra- and inter-class similarities was 0.0690. However, this value in the similarity matrix constructed by iPcc on the first-order correlation features became 0.5226, with an average intra-class similarity of 0.4274 and an average inter-class similarity of −0.0952. By increasing the order of the correlation features (Figure 4), iPcc further highlighted the sample similarity from the same disease class and enlarges the sample distinctiveness from different disease classes. When the order of the correlation features was five, the average intra-class sample similarity was 0.8385 and the average inter-class sample similarity was −0.8413. The difference between the intra- and inter-class sample similarities approached 2.

**Figure 5.**
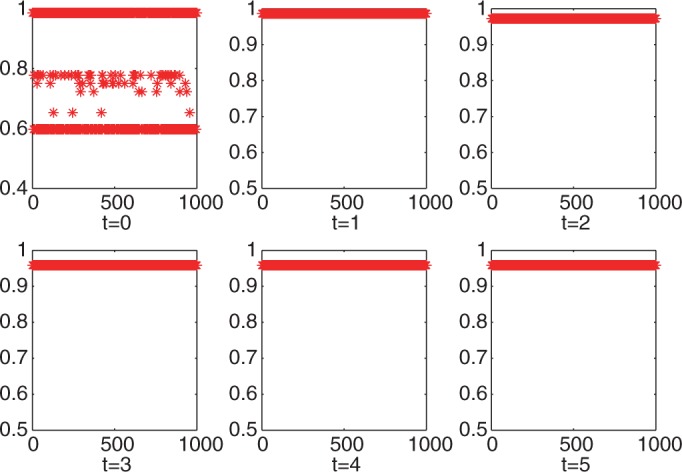
Clustering accuracy of 1000 *k*-means (*k* = 2) runs on the leukemia data set based on the original, first-, second-, third-, fourth- and fifth-order correlation features.

The great power of iPcc was further demonstrated by its big leverage effect on the accuracy and robustness of clustering algorithms, e.g. *k*-means. We ran *k*-means (implemented in Matlab R2011b) 1000 times on the original features to cluster the samples into two groups. Compared with the true disease classes, we found that 357 runs produced accuracy of <60%, 70 runs produced accuracy of between 60 and 90% and 573 runs produced accuracy of >90%. However, 1000 runs of *k*-means on the first-order correlation features constructed by iPcc produced accuracy of >90% (Figure 5). Increasing the order of the correlation features suggested that the accuracy of *k*-means stabilized ∼96%.

**Figure 6.**
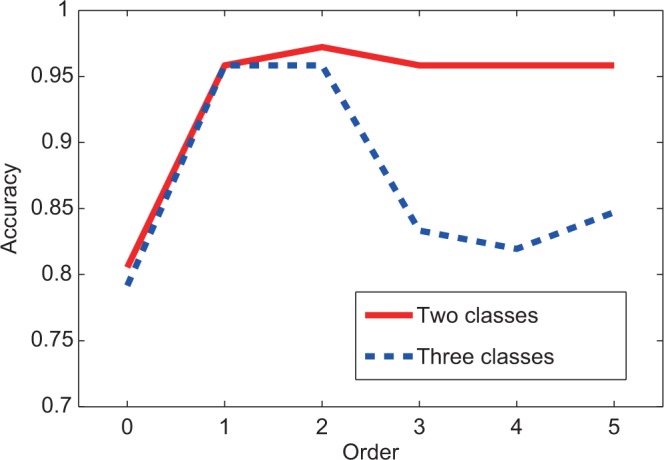
The leave-one-out prediction accuracy by the naïve Bayes classifier on the leukemia data set based on the original, first-, second-, third-, fourth- and fifth-order correlation features.

Besides the clustering algorithm, iPcc also enhanced the classification algorithms, e.g. the naïve Bayes classifier. We evaluated the prediction accuracy of the naïve Bayes classifier on the original features by the leave-one-out cross-evaluation, which yielded 80% accuracy. The accuracy increased to 96% quickly on the first-order correlation features. On the second-order correlation features, the accuracy even approached 97%. Increasing the order further, the accuracy stabilized at 96% (Figure 6).

### iPcc improves the performance of algorithms for disease class discovery and prediction on real prostate cancer data set with multiple classes

**Figure 7.**
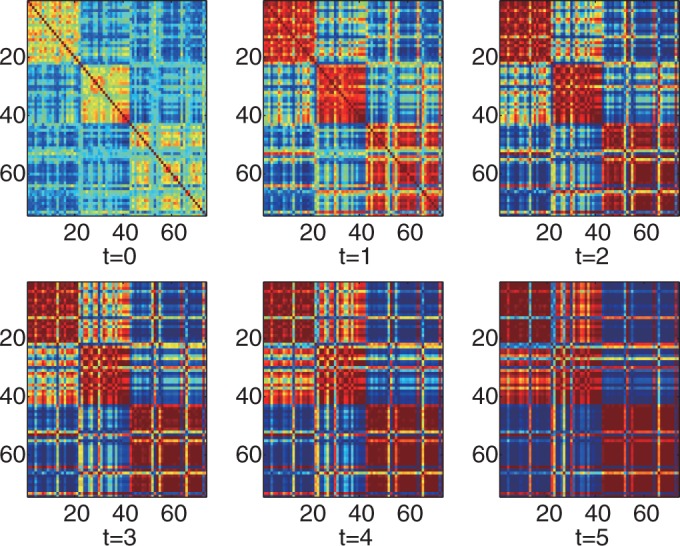
Heatmaps of the prostate cancer sample relationships on the original features, first-, second-, third-, fourth- and fifth-order correlation features. Samples 1–20 are normal samples. Samples 21–42 are metastatic prostate cancer samples, and samples 43–74 are localized prostate cancer samples.

Besides the good performance of iPcc on two-class situations, its great power on multi-class gene expression data sets was also demonstrated. We extracted 22 normal samples, 20 metastatic prostate cancer samples and 32 localized prostate cancer samples from the whole prostate cancer data set, selected the top 1000 informative genes by F-test and constructed a three-class gene expression data set. We constructed the sample similarity matrices based on the original features and the first-, second-, third-, fourth- and fifth-order correlation features (Figure 7). We found that the three-class pattern of samples was highlighted with the order of the correlation features increasing. The difference between the average intra- and inter-class sample similarities on the original features was 0.4028 (intra: 0.2890, inter: −0.1138). However, the difference between the first-order correlation features was 0.8518 (intra: 0.5600, inter: −0.2918). The difference on the second-order correlation features was 0.9962 (intra: 0.6520, inter: −0.3442). The difference approached the maximum on the third-order correlation features (1.006, with intra: 0.6592 and inter: −0.3465). Increasing the order further, the difference decreased a bit because the normal samples and the metastatic prostate cancer samples began to show similar correlation features, i.e. three classes begin to converge to two classes.

Furthermore, we examined the impact of iPcc on the performance of *k*-means clustering. We ran *k*-means 1000 times on the original features, and found that 105 runs generated results with accuracy of <70%. However, all 1000 runs on the first-order correlation features generated results with accuracy of >90%. Runs of 998 and 1000 produced accuracy of >90% on the second- and third-order correlation features, respectively. On the fourth- and fifth-order correlation features, the accuracy of *k*-means oscillated between 60 and 95% because the three classes began to converge into two classes. The leave-one-out accuracy of the naïve Bayes classifier on the original features was 93%, and the accuracy values on the first-, second-, third-, fourth- and fifth-order correlation features were 93, 90, 90, 90 and 90%, respectively.

In fact, the performance of iPcc in multi-class situations was also demonstrated in the leukemia data set in which the ALL samples can be further divided into B-cell ALLs and T-cell ALLs. The difference between the average intra- (AML–AML, B-cell-ALL–B-cell-ALL and T-cell-ALL–T-cell-ALL) and inter-class (AML–B-cell-ALL, AML–T-cell-ALL and B-cell-ALL–T-cell-ALL) sample similarity values based on the original features was 0.0702. However, the differences on the first-, second-, third-, fourth- and fifth-order correlation features were 0.5002, 0.9755, 1.2217, 1.3316 and 1.3567, respectively. In 1000 *k*-means runs on the original features, 130 runs produced accuracy of >80%, while 247, 114, 276 and 1000 runs produced accuracy of >80% on the first-, second-, third- and fourth-order correlation features. On the fifth-order correlations features, *k*-means generated two clusters although *k* was set as three. This is because B-cell ALLs and T-cell ALLs were merged into one cluster in the high-order correlation features. The prediction accuracy of the naïve Bayes classifier on the original features, the first-, second-, third-, fourth- and fifth-order correlation features for three classes was 79, 96, 96, 83, 82 and 85%, respectively.

### iPcc provides a convenient means to deal with batch effects in the real psoriasis data sets

We applied iPcc on the psoriasis data sets to show its performance in situations with batch effects. First, we applied iPcc to examine the sample relationships for GSE13355 and GSE14905 individually. The results suggested that those psoriasis samples were weakly discriminated from the normal samples based on the whole 54 676 original features for both of the two data sets. On greatly reducing the size of the correlation features, the distinctiveness between psoriasis from normal samples was further underlined and confirmed. Clustering by *k*-means on both the original and correlation features of the GSE13355 data set obtained 99% accuracy. For GSE14905, the clustering accuracy by *k*-means was 98% on the original, the first-, second-, fourth- and fifth-order correlation features and 99% on the third-order correlation features. The leave-one-out prediction accuracy of the naïve Bayes classifier for the GSE13355 data set was 96% on the original features, 99% on the first- and second-order correlation features and 98% on the third-, fourth- and fifth-order correlation features. The leave-one-out prediction accuracy of the naïve Bayes classifier for the GSE14905 data set was 96% on the original, first- and second-order correlation features, and 98% on the third-, fourth- and fifth-order correlation features.

**Figure 8.**
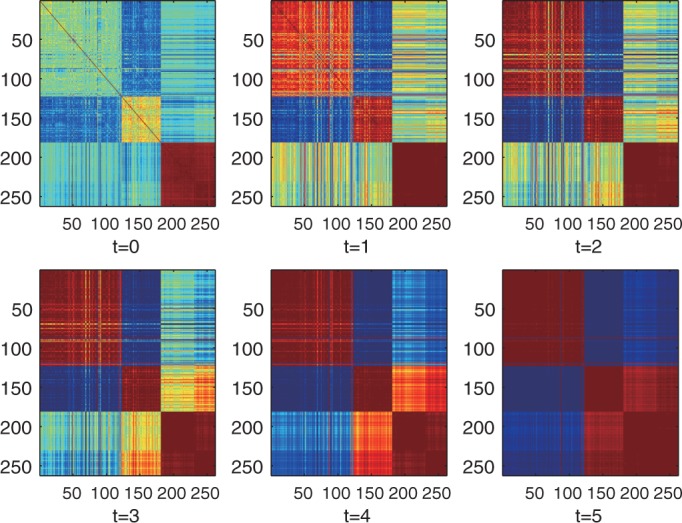
Heatmaps of the psoriasis sample relationships on the original, first-, second-, third-, fourth- and fifth-order correlation features without batch effect removal. Samples 1–122 are normal samples from GSE13355. Samples 123–180 are psoriasis samples from GSE13355. Samples 181–229 are normal samples from GSE14905, and samples 230–262 are psoriasis samples from GSE14905.

However, we observed that the original features suggested that the sample relationship of the combined data set (concatenating after gene-wise normalization) was dominated by three clusters: the normal samples in GSE13355, the psoriasis samples in GSE13355 and all the samples (both normal and psoriasis) in GSE14905. Correlation features constructed by iPcc confirmed the observation based on the original features, and further suggested that the psoriasis samples in GSE13355 were more similar to those samples in GSE14905 (Figure 8). We clustered the samples by k-means on the original features and performed 1000 runs. Five runs obtained accuracy of >90%. One hundred forty-seven runs obtained accuracy of <60%, and 848 runs produced accuracy of between 80 and 90%. On the first-correlation features, the clustering accuracy by *k*-means stabilized at 59%. On the second-, third-, fourth- and fifth-order correlation features, the clustering accuracy by *k*-means stabilized at 81%. The leave-one-out prediction accuracy by the naïve Bayes classifier on the original features was 80%, similar to the clustering accuracy. However, on the first-order correlation features, the leave-one-out prediction accuracy increased to 97%. On the second-order correlation features, the accuracy further increased to 98%. On the third-, fourth- and fifth-order correlation features, the accuracy decreased to 96, 91 and 91%, respectively.

**Figure 9.**
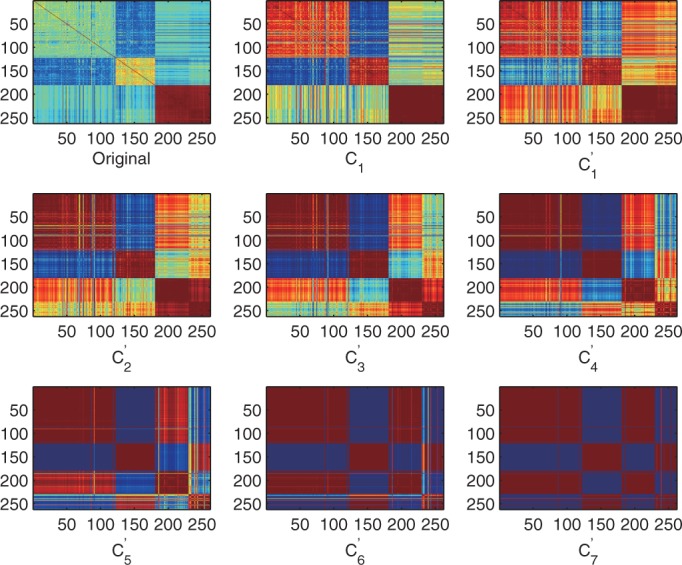
Heatmaps of the psoriasis sample relationships on the original, first-order correlation and batch-effect-removed correlation features. C_1_, the first-order correlation features; C^’^_1_, the updated first-order correlation features with batch effect removal; C^’^_2_, … , C^’^_7_, the correlation features generated based on C^’^_1_. Samples 1–122 are normal samples from GSE13355. Samples 123–180 are psoriasis samples from GSE13355. Samples 181–229 are normal samples from GSE14905. Samples 230–262 are psoriasis samples from GSE14905.

Although the gene-wise normalization step tried to remove batch effects, the above observation suggested that batch effects still influenced the global sample relationship. Here, we show how iPcc removes the batch effects based on correlation features. Because we already knew the experiments were done in two different laboratories, we constructed a sample similarity matrix 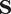 by setting 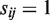 if the experiments of sample *i* and *j* were done in the same laboratory, otherwise setting 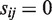 if the experiments of sample *i* and *j* were done in different laboratories. We generated the first-order correlation features 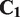 and updated 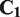 by 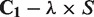, where 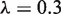 in the real computation. Based on the updated 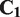, we further constructed the second-, third-, fourth-, fifth-, sixth- and seventh-order correlation features according to iPcc. The sample relationship revealed by the batch-effect-removed features highlighted the similarity among the normal samples and among those psoriasis samples from different laboratories (Figure 9). One thousand runs of *k*-means on the third-, fourth-, fifth- and sixth-order updated correlation features obtained stable 86, 94, 97 and 98% accuracy, significantly larger than the clustering accuracy on the original features or the un-updated correlation features. The leave-one-out prediction accuracy by the naïve Bayes classifier also stabilized at 98% on the fourth-, fifth- and sixth-order updated correlation features.

We compared the batch-effect-removal method by iPcc with feature selection methods, which are frequently used to improve the quality of clustering and prediction. We selected the top 1000 informative genes by F-test and evaluated sample relationship, clustering accuracy and prediction accuracy. The average difference between the intra- and inter-class sample similarities was 0.8592 on the original features. One thousand runs of *k*-means on the original features produced 490 runs with accuracy of 98% and 510 runs with accuracy of 81%. The leave-one-out prediction accuracy of the naïve Bayes classifier on the original features was 98%. Thus, our iPcc-based batch-effect-removal method outperforms, or at least is comparable with, the performance of F-test-based feature selection methods.

By applying iPcc on the 1000 genes selected by F-test, we obtained the intra-class versus inter-class difference values on the first-, second-, third-, fourth- and fifth-order correlation features of 1.2800, 1.3629, 1.4095, 1.4271 and 1.4251, respectively, larger than that on the originally selected genes. One thousand runs of *k*-means on the first-order correlation features yielded 598 runs with an accuracy of 98% and 402 runs with an accuracy of 84%. On the second-order correlation features, the accuracy of 1000 runs of *k*-means stabilized at 97%. The leave-one-out prediction accuracy of the naïve Bayes classifier on the original features and all the correlation features was 98%. Thus, iPcc can also enhance the power of feature-selection methods.

## DISCUSSIONS AND CONCLUSION

With the development of high-throughput technologies, thousands of genes can be measured simultaneously by microarrays or next-generation sequencing technologies. This facilitates comprehensive characterization of the biomedical states of samples but also introduces much irrelevant information. In this study, we proposed a novel feature extraction method, named iPcc, to extract the underlying patterns from noisy data sets through introducing the ‘correlation feature’ concept with iterative Pearson correlation coefficients. Simulations and evaluations on real data sets demonstrate that iPcc greatly improves the disease class discovery and prediction based on the gene expression profiles.

The effectiveness of iPcc may partially originate from reduced dimensionality. In the high-throughput experiments, the number of samples is generally much lower than the number of measured genes, resulting in the ‘curse of dimensionality’. This feature extraction method reduced the dimensionality from thousands of genes to tens of samples. The reduced dimensionality allows rapid and accurate computation of the global optimum of many clustering and classification algorithms and thus improves accuracy. The effectiveness of iPcc may also originate partially from the introduced ‘correlation features’. Without rephrasing the gene expression levels of samples, correlation features introduced by iPcc used the similarity between samples, which is independent of the precise gene expression levels but is related to the trends of the profiles. These features are similar to clinicians’ clinical experience, which is helpful in diagnosis and prognosis. The effectiveness of iPcc is dependent on the measure of Pearson correlation coefficients. We evaluated those ‘correlation features’ computed by the Spearman correlation coefficients, and found that ‘iScc’ does not have the same effect. We will further investigate the differences between Pearson correlation coefficients and Spearman correlation coefficients in the iterative computation setting. The effectiveness of iPcc also relies on rapid convergence (42,43). For the toy, simulation and real data sets, we observed that those values of correlation features quickly converge to 1 or −1 as the order increases. Although the computation of iPcc is rather simple, we have not found a proper mathematical tool to prove the convergence of iPcc owing to the difficulties posed by the specific features of iPcc, including: (i) the input and output of iPcc are structured matrices; (ii) only a recursion formula is available for iPcc now; (iii) the convergence of iPcc occurs at each element, rather than a common norm of the matrix; (iv) the formula is nonlinear; (v) no constraint exists among the elements. We will continue to seek the mathematical proof of the rapid convergence of iPcc in the future.

Because the computation of iPcc does not require sample class information, it is essentially unsupervised. When confounding factors exist, iPcc can also enlarge the biases embedded within the gene expression data set instead of the subject information, just like the batch effects in the psoriasis data set. Thus, it works best on those data sets with irregular noise. However, if the confounding factors are known, iPcc provides an efficient means to remove the effects of those factors. However, iPcc is not an independent algorithm for disease class discovery or prediction. It provides an effective means to underpin the underlying patterns embedded within the gene expression data sets from the feature extraction perspective. Therefore, it can be used in combination with other clustering, classification, feature selection and feature extraction algorithms, as demonstrated in the results section.

We also observed that multiple sample classes could converge to two classes in the high-order correlation feature space. For example, the B-cell ALLs and T-cell ALLs became undistinguishable in the high-order correlation feature space. This is closely related to the class relationship. Because T-cell ALLs are more similar to B-cell ALLs than to AMLs, iPcc merges T-cell ALLs with B-cell ALLs when the iteration number is adequately large. Because iPcc is unsupervised, it would also enlarge noise in high order of correlation features. Therefore, the optimal iteration number of iPcc is dependent on the specific noise level and the information users needed. Because iPcc generates correlation features, a certain order of features can also be jointly used with the original and other-order correlation features.

In conclusion, we proposed a novel feature extraction method that is noise-resistant, i.e. iPcc, based on iterative Pearson correlation coefficients for disease class discovery and prediction based on high-throughput gene expression profiles. Evaluations on both simulated and real gene expression data sets suggest that iPcc not only can highlight the patterns underlying high-dimension gene expression files but also can greatly improve the accuracy of disease class discovery and prediction based on clustering and classification algorithms. This feature extraction method is expected to be a useful tool to boost the development of clinical diagnosis and prognosis.

## FUNDING

Funding for open access charge: National Natural Science Foundation of China [31200106, 11131009 and 61171007].

*Conflict of interest statement.* None declared.
